# Anti-GnRH antibodies can induce castrate levels of testosterone in patients with advanced prostate cancer

**DOI:** 10.1054/bjoc.2000.1315

**Published:** 2000-07-24

**Authors:** M S Simms, D P Scholfield, E Jacobs, D Michaeli, P Broome, J E Humphreys, M C Bishop

**Affiliations:** Department of Urology, City Hospital, Hucknall Rd, Nottingham, NG51PB, UK; Aphton Corporation, PO Box 1049, Wodland, California, CA 95776, USA; Cancer Studies Unit, Department of Surgery, Queen’s Medical Centre, Nottingham, NG72UH, UK

**Keywords:** prostate cancer, hormonal therapy, anti-GnRH antibodies

## Abstract

D17DT consists of the GnRH decapeptide linked to diphtheria toxoid. The aim of this pilot study was to assess the tolerance of D17DT and the production of anti-GnRH antibodies from two doses, 30 and 100 μg, in patients with locally advanced prostate cancer. Twelve patients with histologically proven prostate cancer in whom hormonal therapy was indicated were recruited. Patients received either 30 or 100 μg given intramuscularly on three separate occasions over six weeks. Patients were followed up and blood was taken for estimation of serum testosterone, PSA and anti-GnRH antibody titre. Overall the drug was well tolerated. In 5 patients a significant reduction in serum testosterone and PSA was seen. Castrate levels of testosterone were achieved in 4 and maintained for up to 9 months. Patients with the highest antibody titre had the best response in terms of testosterone suppression. This study shows that it is possible to immunize a patient with prostate cancer against GnRH to induce castrate levels of testosterone. This state appears to be reversible. This novel form of immunotherapy may have advantages over conventional forms of hormonal therapy and further studies are warranted in order to try and increase the proportion of responders. © 2000 Cancer Research Campaign


British Journal of Cancer (2000) 83(4), 443–446
© 2000 Cancer Research Campaign
doi: 10.1054/ bjoc.2000.1315, available online at http://www.idealibrary.com on IDEAL®

### Anti-GnRH antibodies can induce castrate levels of testosterone in patients with advanced prostate cancer

**MS Simms[1], DP Scholfield[1], E Jacobs[3], D Michaeli[2], P Broome[1], JE Humphreys[2] and MC Bishop[1]**

[1]Department of Urology, City Hospital, Hucknall Rd, Nottingham NG51PB UK; [2]Aphton Corporation PO Box 1049, Wodland, California, CA 95776, USA; [3]Cancer Studies Unit, Department of Surgery, Queen's Medical Centre, Nottingham, NG72UH, UK

**Summary** D17DT consists of the GnRH decapeptide linked to diphtheria toxoid. The aim of this pilot study was to assess the tolerance of D17DT and the production of anti-GnRH antibodies from two doses, 30 and 100 μg, in patients with locally advanced prostate cancer. Twelve patients with histologically proven prostate cancer in whom hormonal therapy was indicated were recruited. Patients received either 30 or 100 μg given intramuscularly on three separate occasions over six weeks. Patients were followed up and blood was taken for estimation of serum testosterone, PSA and anti-GnRH antibody titre. Overall the drug was well tolerated. In 5 patients a significant reduction in serum testosterone and PSA was seen. Castrate levels of testosterone were achieved in 4 and maintained for up to 9 months. Patients with the highest antibody titre had the best response in terms of testosterone suppression. This study shows that it is possible to immunize a patient with prostate cancer against GnRH to induce castrate levels of testosterone. This state appears to be reversible. This novel form of immunotherapy may have advantages over conventional forms of hormonal therapy and further studies are warranted in order to try and increase the proportion of responders. © 2000 Cancer Research Campaign

**Keywords:** prostate cancer; hormonal therapy; anti-GnRH antibodies

The work of Huggins and Hodges (Huggins and Hodges, 1941) established that androgen deprivation in patients suffering from prostate cancer results in a favourable response in the majority. Since such treatment is palliative in nature, it has traditionally been reserved for those with either locally advanced or metastatic disease.

Androgen deprivation can be achieved either medically or surgically. Surgical castration is still considered the 'gold standard' endocrine treatment. Medical castration can be achieved by depot LHRH agonists such as goserelin and buserelin. Oral synthetic oestrogens e.g. diethylstilboestrol can also bring about castrate levels of testosterone. In addition steroidal antiandrogens such as cyproterone may be used as monotherapy to reduce serum testosterone and interfere with androgen activity at a cellular level.

D17DT is an immunogen consisting of the GnRH (gonadotrophin releasing hormone) decapetide as a hapten, linked through its amino terminus via a spacer to diphtheria toxoid. The conjugate is formulated as a sterile, milky-white, semi-viscous, water-in-oil injection. In experiments, injection into rabbits has been shown to result in production of anti-GnRH antibodies. In addition, passive immunisation via intraperitoneal injection of anti-GnRH antibodies in nude mice bearing oestrogen sensitive MCF7 human breast cancer xenografts inhibits tumour growth (Jacobs et al, 1999).

As the first study of this vaccine in man, the primary aim of this pilot study was to establish the safety and tolerance of two doses of D17DT, 30 and 100 μg, as an intramuscular injection and to assess production of anti-GnRH antibodies. Secondary endpoints were suppression of serum testosterone and PSA levels.

*Received 23 November 1999*
*Revised 3 April 2000*
*Accepted 13 April 2000*

*Correspondence to:* MS Simms

### MATERIALS AND METHODS

Local Ethics Committee approval was granted for the study. Twelve men with advanced prostate cancer (mean age 75 years) who were suitable for endocrine treatment were recruited. All of these men had locally advanced histologically-proven T3/4 prostate cancer and 1 patient was found during the course of the study to have bony metastases. All patients had a life expectancy of at least 3 months and WHO performance status of 2 or less at the beginning of the study. Informed consent was obtained prior to study commencement.

The first 6 patients recruited received the 30 μg dose and the next six received the 100 μg dose given in three separate doses so that patients received a total dose of either 90 or 300 μg of the drug.

Each patient received 3 i.m. injections of D17DT, given in a volume of 0.2 ml per injection into alternate limbs at weeks 0, 2 and 6. Patients were followed up at 2-weekly intervals for twelve weeks and at 4 weeks thereafter. Subsequent follow-up was determined by response to treatment.

During the course of the study, blood samples were taken for serum PSA (Abbott AxSym), LH (Abbott AxSym) and total testosterone (RIA). Blood was centrifuged within 4 hours of being taken and assay was performed within 24 hours.

Serum was also stored at –20°C for estimation of anti-GnRH antibody titre. An ELISA method was used for this purpose. Serial dilutions of sera were incubated in a 96-well plate which had been coated with GnRH-BSA (this consisted of the specific GnRH peptide conjugated to bovine serum albumin) antigen. Anti-GnRH antibodies bound to the antigen were detected using goat anti-human antibody conjugated to alkaline phosphatase. P-nitrophenyl phosphatase was added as the substrate and the resultant colour change assessed by absorbance at 405 nM. Titres for patient sera

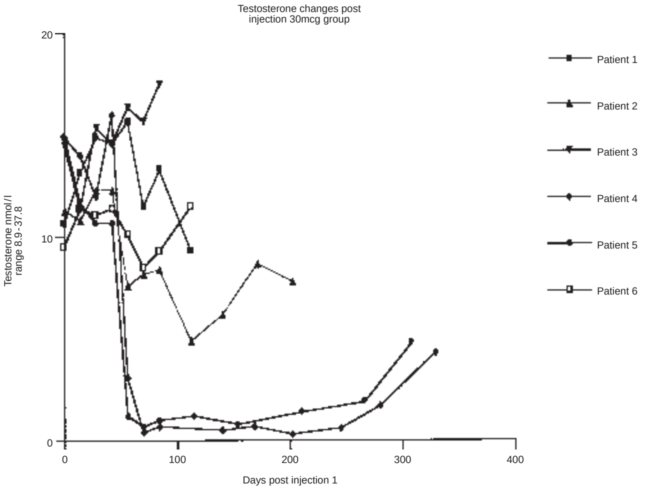

**Figure 1** Testosterone changes post injection (30 µg group)

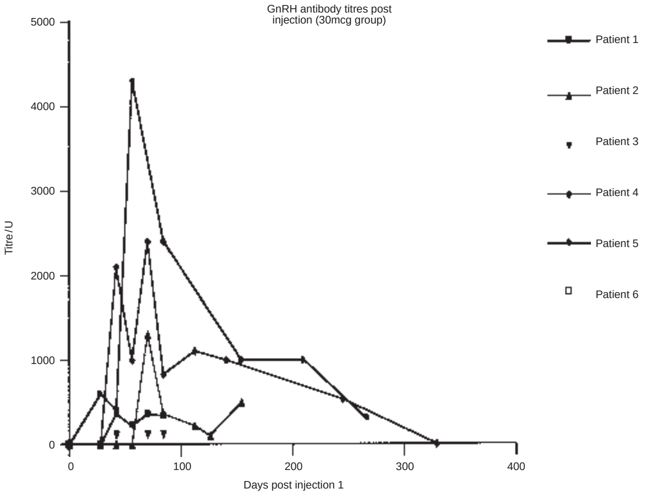

**Figure 3** GnRH antibody titres post injection (30 µg group)

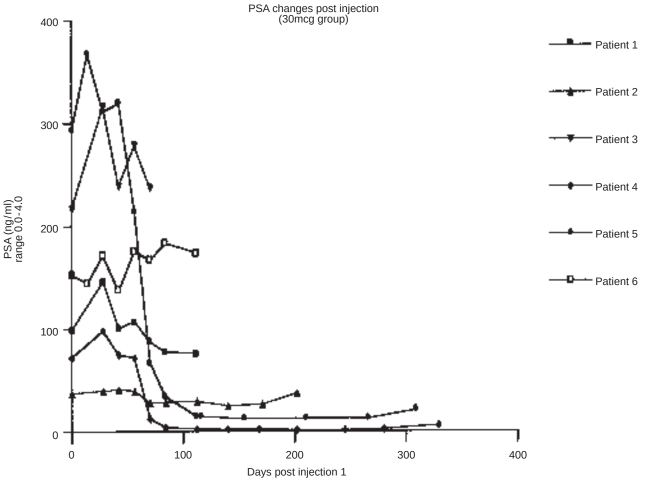

**Figure 2** PSA changes post injection (30 µg group)

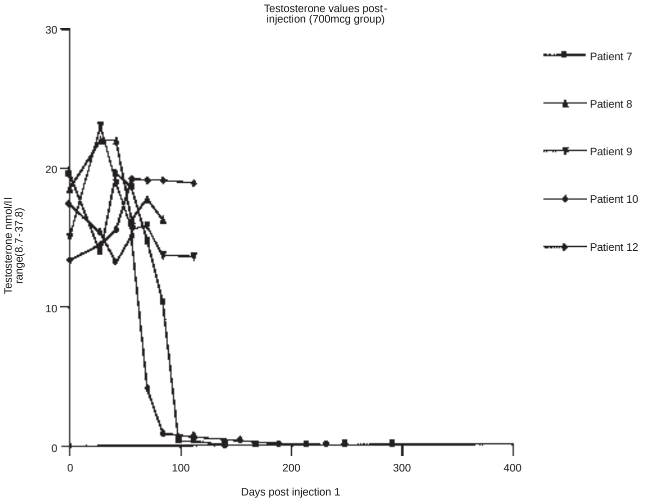

**Figure 4** Testosterone changes postinjection (100 µg group)

were calculated from a standard curve and appropriate controls included in the assay.

During the course of the study any adverse reactions were noted. Attention was paid specifically to the presence of injection site reactions.

### RESULTS

Overall the drug was well tolerated. Adverse events included moderate injection site pain in 9 patients. This lasted between 1 and 11 days post-injection and was not apparent after every injection in these patients. Two patients described mild 'flu'-type symptoms of shivering and headache which lasted up to 2 days on single occasions. These may have been unrelated to the drug.

Prior to immunization all patients had undetectable GnRH antibody titres. Eleven of the 12 patients developed anti-GnRH antibodies during the course of the study, although not all to the same degree (Figures 3 and 6). A marked GnRH antibody titre of over 1000 U (U= arbitrary units) was seen in patients 2, 4, 5, 7 and 12. In the remaining patients, the antibody rise was not as significant and ranged from a maximum titre of between 0 and 400 U.

Immunization with D17DT resulted in significant reduction of serum testosterone in 5 patients (2, 4, 5, 7 and 12). Castrate levels were seen in patients 4, 5, 7 and 12, achieved between 56 and 98 days post injection (Figures 1 and 4). In patient 2 a partial, but clinically significant reduction in testosterone to a minimum value of 4.9 mmol/l was seen 112 days post injection. In all of these patients the decline in serum testosterone seemed to coincide with the peak antibody titre. The fall in serum testosterone was accompanied by fall in LH and FSH. In particular, LH was undetectable in patients 4, 5, 7 and 12, who of course had the best testosterone response.

Patient 11 was recruited to the trial following a TURP (transurethral resection of prostate) which showed a poorly differentiated adenocarcinoma. A subsequent bone scan revealed multiple metastases. At that stage he had received two doses of D17DT and it was felt that conventional therapy was needed. His results are therefore not included in the figures.

Not surprisingly, PSA fell significantly in those patients whose testosterone was suppressed during the course of the study (Figures 2 and 5). Follow up has continued on these patients. In patient 2, who had a partial response, serum testosterone gradually

© 2000 Cancer Research Campaign

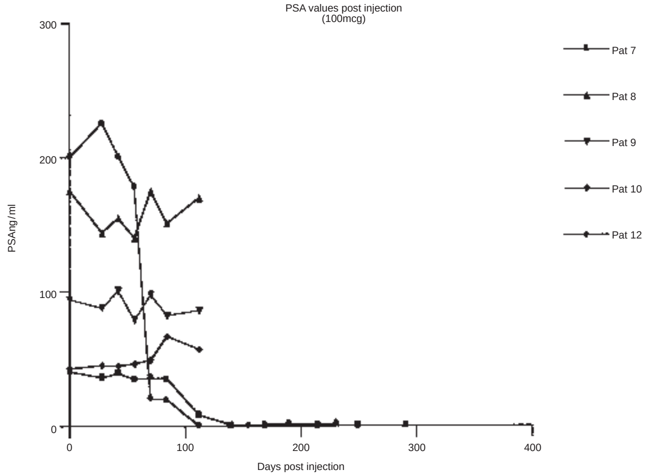

**Figure 5** PSA changes post injection (100 µg group)

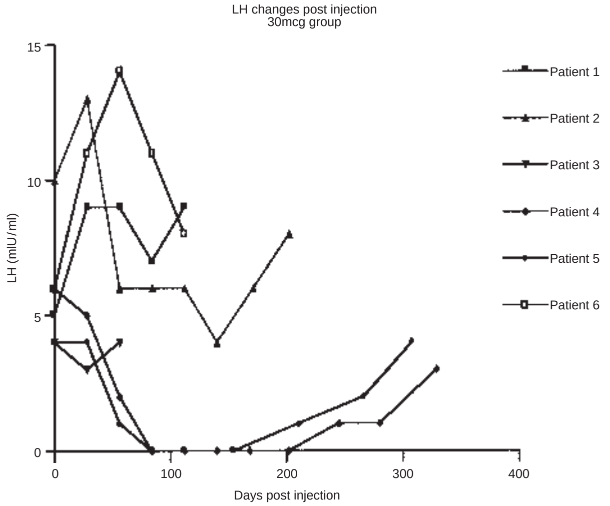

**Figure 7** LH changes post injection (30 µg group)

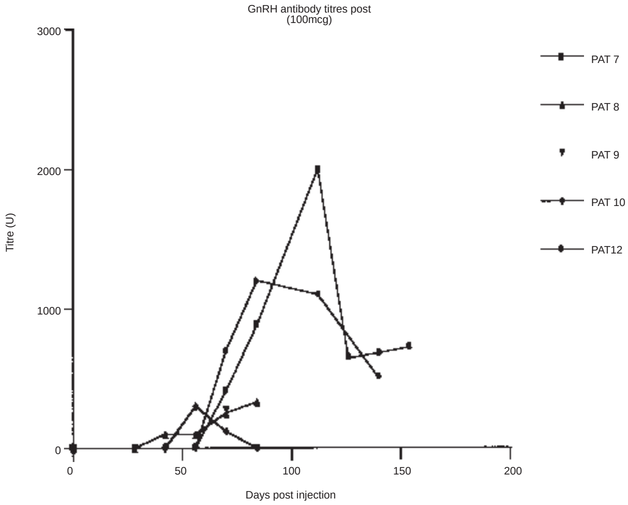

**Figure 6** GnRH antibody titres post injection (100 µg group)

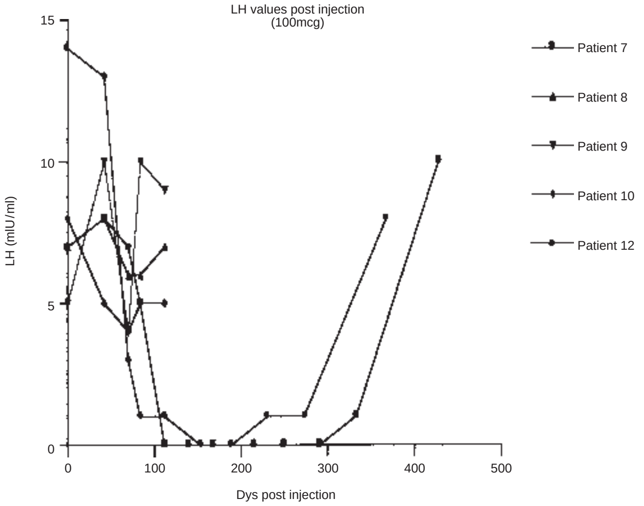

**Figure 8** LH values post injection (100 µg group)

increased back towards the normal range from 168 days post-injection. In patients 4 and 5 (30 µg group) serum testosterone began to rise as antibody titre gradually declined, though castrate levels were maintained for between 8 and 9 months. Follow up continues on the patients in the 100 mcg group who responded (7 and 12). In these patients, serum testosterone remained at castrate levels for 9 months and has now started to return towards the normal range.

### DISCUSSION

This study shows that even a fractional dose of D17DT can elicit a response in some patients and that antibodies raised against a specific hormone (GnRH) can induce a sustained beneficial response in patients with advanced prostate cancer. Previous work in animals has shown that production of anti-GnRH antibodies in response to immunisation causes a decline in testosterone and testicular atrophy in rodents (Jayashankar et al, 1989). However, experience in humans is limited (Talwar, 1997). A similar type of vaccine has been shown to inhibit gonadotrophins in post-menopausal women (Gual et al, 1997). This paper demonstrates 'proof of principle' in patients with prostate cancer. This novel form of immunotherapy has been described recently in a patient with refractory hypercalcaemia from metastatic parathyroid carcinoma (Bradwell and Harvey, 1999). The patient was immunised against her own parathyroid hormone, with a resultant fall in serum calcium and clinical improvement.

The production of antibodies to GnRH is a complex process (Stevenson, 1999) and depends on successful antigen uptake and presentation to T and B lymphocytes. Immunization with D17DT will result in the production of antibodies both to DT and to GnRH. In this study the anti-GnRH antibody titre was measured specifically by ELISA. A previous paper has shown that passive immunisation of female nude mice with affinity purified anti-GnRH antibodies produced from the serum of rabbits immunised with D17DT resulted in atrophy of reproductive organs (Jacobs et al, 1999). The authors showed that passive immunisation with anti-GnRH antibodies resulted in a significant fall in serum LH and oestrogen compared with controls. This provides evidence for suppression of pituitary gonadal function by anti-GnRH antibodies. In addition growth of the oestrogen sensitive MCF7 breast cancer xenograft in these mice was inhibited by administration of -

© 2000 Cancer Research Campaign anti-GnRH antibodies. The amount of GnRH in D17DT is extremely small and data from early work with LHRH agonists (Walker et al, 1984) indicate that this dose would be very unlikely to produce testosterone suppression to castrate levels. LH levels were seen to increase slightly during the first few weeks of treatment in some of the patients (Figures 7 and 8). Interestingly however a rise was not seen in 3 of the 4 patients who developed castrate levels of testosterone (in patient 7 there was a very slight increase in serum LH from 7 to 8 mIU/ml) and this too would support the fact that the GnRH effect in producing a castrate state is negligible.

It seems from this study that the clinical response is related quantitatively to antibody production. The patients with the highest anti-GnRH antibody titres had the best response in terms of testosterone and PSA suppression. Work in animals has shown that the antibody response is dependent on many factors including dose of drug, number of injections and the choice of adjuvant and carrier (Ferro and Stimson, 1996). Epitope suppression is believed to occur when a host is exposed to an immunogenic dose of carrier protein, followed by immunization with a hapten conjugated to the same or similar carrier molecules. The anti-hapten antibody response is thereby suppressed and ineffectual immunisation may occur. Since immunization against diphtheria was introduced into the UK in 1940, most of the patients in this study will have been exposed to the carrier protein prior to the commencement of this trial. This may partly explain why all patients did not respond to the same degree. However, it has been shown that the effects of epitope suppression can be overcome by increasing the number of doses in the immunisation regimen (Ferro and Stimson, 1996). In addition it has been suggested that carrier pre-sensitisation (i.e. pre-immunisation with diphtheria toxoid) may have the advantage of adjuvant free administration. Since adjuvants have the potential for causing a degree of tissue damage this would be desirable. This form of immunotherapy may also be applicable to other hormone sensitive forms of cancer, such as carcinoma of the breast.

Both 30 and 100 μg can be regarded as low doses, mainly intended to assess tolerance rather than determine efficacy. In terms of therapeutic efficacy, there did not appear to be a great deal of difference between the 30 and 100 mcg dose. Both doses produce castrate levels of testosterone lasting up to 9 months.

D17DT may have advantages over more conventional forms of hormonal treatment in terms of ease of administration, theoretical lack of hormonal flare and length of testosterone suppression. In order to investigate the precise effect of D17DT on hormonal flare it will be necessary to observe LH and testosterone levels more closely during the initial 2 weeks of treatment. One potential disadvantage of this treatment is the fact that it can take up to 3 months for castrate levels of testosterone to develop. The role of hormonal therapy however in prostate cancer is set to increase. Traditionally hormonal therapy has been delayed until the onset of significant clinical symptoms and the decision to treat has been based on a balance between therapeutic advantages and side-effects from hormonal therapy. Recent studies have suggested some degree of advantage for early androgen withdrawal when compared to deferred therapy (Kirk et al, 1996) and many patients who will be started on hormonal therapy in the future will in fact be asymptomatic.

Intermittent endocrine therapy (Akakura et al, 1993) has been proposed as a form of treatment that may be equal to continuous treatment, and yet may give an enhanced quality of life for patients. This form of therapy may produce a favourable biological tumour effect (Bruchovsky et al, 1990) in that the cyclic nature of this treatment could theoretically prolong the androgen dependent state of a population of cancer cells. It appears that immunotherapy with D17DT is reversible and therefore D17DT could achieve the benefit of cyclic treatment. Further studies will need to investigate the effect of 'booster doses' on testosterone suppression.

High affinity GnRH receptors are found in a high proportion of prostate cancers (Halmos et al, 2000). The role of these receptors in malignancy is not fully understood, although there is evidence suggesting that blocking these receptors in nude mice bearing androgen insensitive prostate cancer xenografts may lead to an inhibition of tumour growth (Jungwirth et al, 1997). D17DT works mainly around the hypothalamic-pituitary axis. It is possible however that anti-GnRH antibodies may also have a direct effect on prostate tumour by affecting GnRH binding to receptors.

In conclusion we have shown that the induction of antibodies to GnRH can produce lasting, castrate levels of testosterone in men with prostate cancer. Future studies will need to address factors in order to assess whether this novel form of therapy can produce reliable and predictable castration in the majority of patients.

### REFERENCES

Akakura K, Bruchovsky N, Goldenberg SL, Rennie PS, Ruckley AR and Sullivan LD (1993) Effects of intermittent androgen suppression on androgen-dependent tumours: apoptosis and serum prostate-specific antigen. *Cancer* **71**: 2782–2790

Bradwell AR and Harvey TC (1999) Control of hypercalcaemia of parathroid carcinoma by immunisation. *Lancet* **353**: 370–373

Bruchovsky N, Rennie PS, Coldman AJ, Goldenberg SL, To M and Lawson D (1990) Effects of androgen withdrawal on the stem cell composition of the Shionogi carcinoma. *Cancer Res* **50**: 2275–2282

Ferro VA and Stimson WH (1996) Effects of adjuvant, dose and carrier presensitisation on the immunisation efficacy of a GnRH analogue. *Drug Des and Discov* **14**: 179–195

Gual G, Garza-Flores J, Menjivar M, Guitierrez-Najar A, Pal R and Talwar G (1997) Ability of an anti-luteinizing hormone-releasing vaccine to inhibit gonadotropins in postmenopausal women. *Fert and Steril* **67**: 404–407

Guinan P, Toronchi E, Shaw M, Crispin R and Sharifi R (1982) Bacillus Calmette-Guerin (BCG) adjuvant therapy in stage D prostate cancer. *Urology* **20**: 401–403

Halmos G, Arencibia JM, Schally AV, Davis R and Bostwick DG (2000) High incidence of luteinising hormone releasing hormone (LHRH) and LHRH receptor gene expression in human prostate cancers. *J Urol* **163**: 623–629

Huggins C, Hodges C (1941) Studies on prostatic cancer. The effect of castration, of estrogen and of androgen injection on serum phosphatases in metastatic carcinoma of the prostate. *Cancer Res* **1**: 293–297

Jacobs E, Watson SA, Michaeli D, Ellis IO and Robertson JF (1999) Anti-gonadotrophin releasing hormone antibodies inhibit the growth of MCF7 human breast cancer xenografts. *Br J Cancer* **80**(3–4): 352–359

Jayashankar R, Chaudhuri MK, Singh O, Alam A and Talwar GP (1989) Semisynthetic vaccine causing atrophy of the prostate. *Prostate* **14**: 3–11

Jungwirth A, Pinski J, Galvan G, Halmos G, Szepeshazi K, Cai RZ, Groot K, Vadillo-Buenfil M and Schally AV (1997) Inhibition of growth of androgen-independent DU-145 prostate cancer in vivo by luteinising hormone-releasing hormone antagonist Cetrorelix and bombesin antagonists RC-3940-II and RC-3950-II. *Eur J Cancer* **33**: 1141–1148.

Kirk D for Medical Research Council Prostate Cancer Working Party Investigators (1997) Immediate versus deferred treatment for advanced prostatic cancer: initial results of the Medical Research Council Trial. *Br J Urol* **79**: 235–246

Stevenson GT (1999) Immunotherapy of non metastatic complication of malignant disease *Lancet* **353**: 340

Talwar GP (1997) Vaccines for control of fertility and hormone-dependent cancers. *Imm and Cell Bio* **75(2)**: 184–9

Walker KJ, Turkes AO, Turkes A, Zwink R, Beacock C, Buck AC, Peeling WB and Griffiths K (1984) Treatment of patients with advanced cancer of the prostate using a slow-release (depot) formulation of the LHRH agonist ICI 118630 (Zoladex®). *J Endocrinol* **103**: R1–R4

© 2000 Cancer Research Campaign
